# Human papillomavirus 16 E6 oncoprotein promotes up-regulation of RNA-binding protein Sam68 in head and neck cancer

**DOI:** 10.3389/fmicb.2026.1657818

**Published:** 2026-02-05

**Authors:** Andrea Cerasuolo, Tiziana Pecchillo Cimmino, Salvatore Gagliarde, Petra Claudia Camilla D’Orsi, Noemy Starita, Sara Amiranda, Anna Lucia Tornesello, Luisa Dassi, Patrizia Bonelli, Franca Maria Tuccillo, Franco Maria Buonaguro, Francesco Longo, Francesco Perri, Rosalia Anna Rega, Rossella De Cecio, Gerardo Ferrara, Franco Ionna, Maria Lina Tornesello

**Affiliations:** 1 Molecular Biology and Viral Oncology Unit, Istituto Nazionale Tumori-IRCCS Fondazione G. Pascale, Naples, Italy; 2 Scuola Superiore Meridionale, Naples, Italy; 3 Innovative Immunological Models Unit, Istituto Nazionale Tumori-IRCCS Fondazione G. Pascale, Naples, Italy; 4 Maxillofacial and ENT Surgery Unit, Istituto Nazionale Tumori-IRCCS Fondazione G. Pascale, Naples, Italy; 5 Medical and Experimental Head and Neck Oncology Unit, Istituto Nazionale Tumori-IRCCS Fondazione G. Pascale, Naples, Italy; 6 Pathology Unit, Istituto Nazionale Tumori-IRCCS Fondazione G. Pascale, Naples, Italy

**Keywords:** head and neck squamous cell carcinoma, HNSCC, HPV, HPV16 E6, HPV16 E6*I, human papillomavirus, OPSCC, SAM68

## Abstract

Head and neck squamous cell carcinoma (HNSCC) are a heterogeneous group of tumors linked to diverse risk factors, such as tobacco and alcohol use, as well as human papillomavirus (HPV) infection. HPV16 E6 and E7 oncoproteins are the main players of cell transformation, with the E6*I isoform increasing during neoplastic progression. The aim of this study was to evaluate the role of cellular splicing factors in the production of E6*I in HPV16-related HNSCC. We have evaluated the levels of splicing factors mRNA (HNRNPA1, HNRNPA2B1, SRSF1, SRSF2, SRSF3, BRM and SAM68) as well as HPV16 E6 and E6*I mRNAs by qPCR in HNSCC as well as in SCC152 and SCC154 cell lines. Overall, 42.4% of HNSCC tested positive for HPV16 DNA, and among these 54% expressed E6*I mRNA. The SRSF3, BRM and SAM68 transcripts were significantly higher in HPV-positive compared to HPV-negative HNSCC (*p* < 0.05), and SAM68 with HPV16 E6*I transcripts concordantly high in both HNSCC and cell lines (*r* = 0.7). Transduction of LXSN_E6 in HPV-negative PCA5 cell line induced production of E6*I mRNA and overexpression of Sam68 protein. In addition, silencing of SAM68 in SCC152 caused decrease of E6*I RNA and reduced cell growth at 48 hr after siRNA transfection. Higher expression of splicing factors in association with HPV was also confirmed in HNSCC TCGA dataset. In conclusion, our results suggest an interplay between the splicing machinery and HPV16 E6*I in HNSCC. These new observations are crucial for the development of novel therapeutic strategies based on SAM68 inhibitors.

## 1 Introduction

Head and neck squamous cell carcinoma (HNSCC) comprises a heterogeneous group of tumors arising from the mucosal epithelium of lip and oral cavity, nasopharynx, oropharynx, hypopharynx and larynx (Johnson et al., 2020). They constitute the sixth most common cancer type in the world with 891’453 new cases diagnosed in 2022 (Bray et al., 2024).

Oral cavity and larynx SCC are mainly associated with tobacco consumption and alcohol abuse, whereas oropharynx SCC are mostly attributed to human papillomavirus (HPV) infection, with large predominance of HPV16 genotype, in European and North American countries (Faraji et al., 2019; Lechner et al., 2022; Stein et al., 2015).

The E6 and E7 oncoproteins encoded by high-risk HPV genotypes are the main players of cell transformation for their ability to cause p53 and pRb degradation, respectively, leading to abnormal cell proliferation (Scheffner et al., 1993; Dyson et al., 1989). In addition, E6 and E7 have been shown to activate many pathways involved in cell malignant transformation, such as angiogenesis, invasion, metastasis and unrestricted telomerase activity (Peng et al., 2024; Tornesello et al., 2018; Tornesello et al., 2023).

Alternative splicing, consisting in the removal of introns from pre-mRNAs through the usage of various combinations of splice donor and acceptor sites, followed by the joining of exons, is the main mechanism of HPV post-transcriptional regulation (Graham and Faizo, 2017; Wu et al., 2017). The HPV16 E6 and E7 oncogenes are transcribed as bi-cistronic pre-mRNAs, subsequently spliced into several mature isoforms, including E6*I, which are differentially produced during the carcinogenic process. In particular, E6*I is the most abundant isoform in HPV-related cervical cancers and HNSCC (Olmedo-Nieva et al., 2018; Ajiro and Zheng, 2015; Cerasuolo et al., 2017; Lin et al., 2015). The E6*I isoform is mainly translated into the E7 oncoprotein and short E6* peptides, which have been recently demonstrated to disrupt the mitochondrial activities and to induce ROS production, thus contributing to the oncogenic activity of high risk HPVs (Tang et al., 2006; Filippova et al., 2014; Williams et al., 2014; Evans et al., 2016).

The fine tuning of the splicing process is mediated by the cellular major spliceosome complex, consisting of five uridine-rich small nuclear RNAs (U1, U2, U4, U5, and U6) and over 100 snRNA associated peptides, together with several RNA binding proteins, such as serine/arginine-rich splicing factors (SRSFs) and heterogeneous ribonucleoproteins (hnRNPs) (Graham and Faizo, 2017; Papasaikas and Valcárcel, 2016; Kastner et al., 2019). SRSFs generally bind to exonic and intronic splicing enhancers and favor the spliceosome assembly, while hnRNPs usually bind to exonic and intronic splicing silencers and inhibit splice factors recruitment on cognate sites (Chen and Manley, 2009; Gehring and Roignant, 2021).

Different studies in cervical cancer showed that splicing factors HNRNPA1, HNRNPA2B1, SRSF1, SRSF2, and SRSF3 are able to bind to HPV16 E6/E7 pre-mRNAs, regulating the production of the E6 full-lenght mRNA as well as of the E6*I isoform during carcinogenesis (McFarlane et al., 2015; Rush et al., 2005; Cheunim et al., 2008; Li et al., 2013). Other RNA-binding proteins, including Brm (Brahma) and Sam68 (Src-associated during mitosis, 68 kDa), which are mainly involved in chromatin remodeling and RNA metabolism, have also been found to regulate the E6/E7 pre-mRNAs processing (Rosenberger et al., 2010).

HPV proteins have been shown to directly regulate the activity of splicing factors (Cerasuolo et al., 2020). For instance, the E2 protein has been shown to possess SRSF-like activity, being able to bind E6/E7 pre-mRNAs and to interact with SRSF4, SRSF5, SRSF6, and SRSF9, causing the exclusion of E6 intron (Graham, 2016; Bodaghi et al., 2009). In addition, the E2 was also shown to bind to SRSF3 promoter, causing SRSF3 increased expression in undifferentiated keratinocytes (Klymenko et al., 2016). The HPV16 E6 oncoprotein was shown to bind the E6/E7 pre-mRNAs and to interact with SRSF4, SRSF6, SRSF9, causing E6 intron retention (Liu et al., 2018).

Dysregulation of the splicing process has been described in different cancer types, causing the production of aberrant mRNA isoforms that promote cell proliferation and migration, drug resistance and reduced apoptosis (El Marabti and Younis, 2018; Zhang et al., 2019). The main cause of splicing alteration is the overexpression of splicing factors, some of which were demonstrated to have direct transforming activity. For example, up-regulation of HNRNPA2B1 in NIH-3T3 mouse cell line enhanced colony formation *in vitro* and tumor growth when injected in mice (Golan-Gerstl et al., 2011). Overexpression of SRSF1 was shown to cause NIH-3T3 cell line transformation through the production of RPS6KB1 oncogenic isoform-2, which causes enhanced cell proliferation *in vitro* and *in vivo* (Karni et al., 2007). Similarly, the ectopic expression of SRSF3 in NIH-3T3 cells increased growth rate *in vitro* and *in vivo*, while SRSF3 silencing in U2OS and HeLa cell lines reduced cell proliferation by blocking cell cycle in G2/M phase (Jia et al., 2010).

The role of splicing factors in HPV E6/E7 pre-mRNAs processing and in HNSCC carcinogenesis has been poorly studied, and the interplay between HPV and splicing factors has not been yet investigated (Biselli-Chicote et al., 2017; Lu et al., 2014; Kang et al., 2009; Ting et al., 2009).

In this study, the expression of splicing factors HNRNPA1, HNRNPA2B1, SRSF1, SRSF2, SRSF3, BRM, SAM68 and of HPV16 E6 full-length and E6*I mRNAs was analyzed in HPV-related HNSCC. Moreover, the role of such splicing factors in E6*I production and the possible interplay with HPV16 oncoproteins was investigated via retroviral transduction of HPV16 E6 in HPV-negative PCA5 SCC cell line and silencing of SAM68 by siRNA transfection in HPV16-positive SCC152 cell line.

## 2 Materials and methods

### 2.1 Patients and samples

The study included 41 participants of whom 33 patients were diagnosed with HNSCC comprising 19 oropharyngeal SCC (OPSCC), eight oral cavity SCC (OCSCC), five laryngeal SCC (LSCC) and one hypopharyngeal SCC (HPSCC). The remaining eight cases were diagnosed with head and neck dysplasia (HND) and were included in the study as control samples. All patients attended the Maxillofacial and ENT Surgery Unit at the Istituto Nazionale Tumori IRCCS Fondazione G. Pascale. Each biopsy was divided in two sections: one was used for histopathological examinations, while the other was stored at −80°C in RNAlater Stabilization Solution (Thermo Fisher Scientific, Waltham, Massachusetts) and subsequently used for the molecular analyses.

SCC-derived HPV-negative PCA5 and CAL27 cell lines as well as HPV16-positive SCC152 and SCC154 cell lines were cultured in Dulbecco’s modified Eagle’s medium (DMEM) with 10% Fetal bovine serum (FBS), 1% L-glutamine and 1% penicillin/streptomycin antibiotic in 5% CO_2_.

The study was conducted in accordance with the Declaration of Helsinki, and approved by the Institutional Review Board and Ethics Committee of Istituto Nazionale Tumori IRCCS Fondazione G. Pascale (authorization number n. 30/22 and n.9/23). The patients provided written informed consent to participate to the study.

### 2.2 DNA and RNA extraction

Genomic DNA was extracted according to previously published method (Dassi et al., 2020). Briefly, 10 mg of tissue samples were digested with proteinase K (150μg/mL at 37°C overnight) in 100μL of lysis buffer (10 mM Tris–HCl pH 7.6, 5 mM EDTA, 150 mM NaCl, 1% SDS), then the DNA isolation was performed with phenol-chloroform-isoamyl alcohol (25:24:1) extraction and ethanol precipitation in 0.3 M sodium acetate (pH 4.6). The DNA quality and quantity was assessed by Nanodrop 2000c (Thermo Fisher Scientific) by calculating the 260 nm/280 nm and 260 nm/230 nm absorbance ratio. All the sample with a 260 nm/280 nm ratio between 1.8 and 2.0 were considered of good quality and included in the analyses.

For total RNA extraction, about 30 mg of tissue samples were dissociated with gentle MACS Octo Dissociator (Miltenyi Biotec, Bergisch Gladbach, Germany). Then, RNA was isolated from all the samples by using RNeasy MiniKit (Qiagen, Hilden, Germany) according to manufacturer procedure. The RNA quality and quantity was assessed as described for DNA.

### 2.3 HPV detection, genotyping, and HPV16 viral load quantification

The integrity of extracted DNA was assessed by PCR amplification of a 134 bp fragment within TP53 gene exon 7, using primers reported in Supplementary Table 1 (Hensel et al., 1991). Then, broad spectrum nested PCR was performed, according to a validated WHO protocol for the detection of alpha HPVs (Eklund et al., 2010). Specifically, 300 ng of genomic DNA were amplified with MY09/MY11 primer pairs (Resnick et al., 1990) for the outer reaction and with MGP primer set for the inner reaction (Söderlund-Strand et al., 2009), containing 5μL of outer reaction, as previously described (Dassi et al., 2020). The amplification reactions were verified by electrophoresis on a 7% polyacrylamide gel, followed by ethidium bromide staining and image analysis using the Gel Doc imaging system (Bio-Rad, Hercules, California).

The HPV genotypes were identified by direct automated DNA sequencing analysis of the amplified products using the primer GP5+ (de Roda Husman et al., 1994) at Eurofins Genomics GmbH (Ebersberg, Germany) and alignments of HPV sequences with those present in the GenBank database using the BLASTn software.^1^

HPV16 viral load quantification was performed by droplet digital PCR (ddPCR). The Minimum Information for Publication of Quantitative Digital PCR Experiments for 2020 (dMIQE2020) checklist was followed for reactions setup (Supplementary Table 2). Briefly, the amplification of HPV16 E6 gene was performed in a 20μL reaction mixture including 10μL of 1X QX200 ddPCR EVAGreen Supermix (Bio-Rad), 300 nM each of E6 specific forward and reverse primers (Supplementary Table 1), 2 μL of DNA and nuclease-free water. The 20 μL reaction mixtures were loaded into DG8 Cartridges (Bio-Rad) with 70 μL of QX200 Droplet Generation Oil for EvaGreen (Bio-Rad) for automatic droplet generation using the QX200 Droplet Generator (Bio-Rad). Droplets were transferred into 96-well plates, which were heat sealed using the PX1 PCR Plate Sealer (Bio-Rad). Amplification was performed in duplicate in a CFX96 thermal cycler (Bio-Rad) and the droplets were read by a QX200 Droplet Reader (Bio-Rad). Finally, data were analyzed with QuantaSoft software version 1.7 (Bio-Rad). TP53 exon 7 was also amplified with specific primers (Supplementary Table 1). The viral copy number was calculated by normalizing the HPV16 E6 gene copy number against the amount of cellular DNA (TP53 gene) with the following formula: viral copy number/GE = number of E6 copies/(number of TP53 copies/2). Limit of blank (LOB) was assessed by amplification of HPV-negative NTERA2, HT3, and PCA23 cell lines DNA (Supplementary Figure 1). Limit of detection (LOD) of the reactions was determined by amplification of 1:10 serial dilutions of SiHa cell line DNA (Supplementary Figure 2).

### 2.4 Splicing factors, HPV16 E6 and E6*I expression analysis by qPCR

A total of 250 ng of RNA for each sample was reverse transcribed by using the iScript cDNA Synthesis Kit (Bio-Rad) in a 20 μL volume reaction containing 1 μL of iScript reverse transcriptase, 4 μL of 5X iScript reaction mix and nuclease-free water. The reaction was incubated in a Mastercycler X50s (Eppendorf, Hamburg, Germany) thermal cycler at 25°C for 5 min and 46°C for 20 min, then the enzyme was inactivated at 95°C for 1 min.

The HNRNPA1, HNRNPA2B1, SRSF1, SRSF2, SRSF3, BRM, and SAM68 splicing factors transcripts were amplified in all samples and cell lines, while the E6 and E6*I transcripts were amplified in HPV16-positive samples as well as in cell lines by qPCR using specific primer pairs reported in Supplementary Table 1. The amplification mixture included 10 μL of 1X Sso Advanced Universal SYBR Green Supermix (Bio-Rad), 10 pmol of each primer, 2 μL of cDNA and nuclease-free water in a final volume of 20μL. The reactions were performed in duplicate with the CFX96 real time PCR Detection System (Bio-Rad).

Gene expression levels were normalized with the 2^–Δ^*^Ct^* method using GAPDH and ACTB as reference genes. Fold changes were calculated with the ΔΔCt method. All Ct values were corrected for primer pairs efficiency, which was calculated generating standard curves of SiHa cDNA serial dilutions.

### 2.5 HPV16 E6 and E6*I expression analysis by ddPCR

The amplification of HPV16 E6 and E6*I mRNAs was performed in a 20 μL reaction mixture including 10 μL of 1X QX200 ddPCR EVAGreen Supermix (Bio-Rad), 200 nM each of E6 and E6*I specific forward and revers primers, 2 μL of cDNA and nuclease-free water (Supplementary Tables 1, 2). The annealing temperature of E6 and E6*I primers was optimized by amplifying SiHa cell line cDNA with a temperature gradient ranging from 52 to 62.8°C for E6 and from 46 to 56°C for E6*I. The optimal annealing temperature was 52°C for both primer pairs. LOB was assessed by amplification of HPV-negative NTERA2, HT3, and PCA23 cell lines cDNA (Supplementary Figure 1). LOD was determined by amplification of 1:2 serial dilutions of SiHa cell line cDNA into HPV-negative NTERA2 cell line cDNA (Supplementary Figure 2).

### 2.6 HPV16 E6 cell line transduction

The PCA5 cell line was stably transduced with empty LXSN retroviral vector, LXSN carrying the HPV16 E6 ORF (LXSN_E6) and LXSN carrying HPV16 E6/E7 ORFs (LXSN_E6E7) using Lipofectamine 2000 reagent (Thermo Fisher Scientific), following the manufacturer’s instruction. Briefly, the Psi2 retrovirus packaging cell line was transduced with 10 μg of pLXSN empty vector, pLXSN_E6 and pLXSN_E6E7 DNA following the manufacturer’s instructions. Then, the medium containing the virions produced by Psi2 cells was collected, filtered with 0.22 μm filters and used for the transduction of PA317 packaging cell line with 4 μg/mL polybrene (Sigma-Aldrich, St. Louis, Missouri, United States). After selection of transduced PA317 cells with 600 μg/mL G418 (Thermo Fisher Scientific), the medium containing the viral particles was collected, filtered with 0.22 μm filters and used for the stable transduction of PCA5 cell line.

### 2.7 SAM68 silencing and cell viability assay

SCC152 cells were seeded in 6-well plates at 3 × 10^5^ cells/well for 24 h and then transfected with 10 μM of siRNAs targeting SAM68 (siSAM68) (Tichon et al., 2018), using Lipofectamine RNAiMAX Reagent (Thermo Fisher Scientific) following the manufacturer’s instructions. The siRNAs used were the following: siSAM68_1 (5′-CAUAAGAACAUGAAACUGA-3′), siSAM68_2 (5′-GCACCCAUAUGGACGUUAU-3′), siSAM68_3 (5′-UAUGAUGGAUGAUAUCUGU-3′) and siSAM68_4 (5′-ACAAGGGAAUACAAUCAAA-3′). In addition, cells were transfected with non-targeting siRNA (siNC, 5′-UUCUCCGAACGUGUCACGU-3′) (Yan et al., 2019). Untransfected cells (blank) were used as control. In addition, cell viability was evaluated at the time of silencing as well as 24 and 48 h after transfection by counting live cells stained with 0.4% Trypan blue (Thermo Fisher Scientific) using the Luna-II automated cell counter (Logos Biosystems, Anyang-si, Gyeonggi-do, South Korea). All the experiments were performed in triplicate.

### 2.8 Protein extraction and western blot

Whole cell protein extracts were obtained from PCA5 cell line transduced with LXSN, LXSN_E6 and LXSN_E6E7 vectors as well as from SCC152 cell line transfected with siSAM68 by using RIPA Lysis Buffer System (Santa Cruz Biotechnology, Texas, United States) and quantified by Bio-Rad Protein Assay (Bio-Rad). Sam68 protein expression was analyzed by western blot. Briefly, 60 μg proteins were separated on 4–15% Mini-PROTEAN TGX precast protein gels (Bio-Rad) and transferred to Amersham™ Hybond P 0.45 PVDF blotting membrane (GE Healthcare, Illinois, United States), then mouse anti-Sam68 primary antibody (Santa Cruz Biotechnology, RRID: not available, sc-514468, 1:3,000 dilution) was incubated overnight at 4°C. Secondary anti-mouse IgG conjugated to horseradish peroxidase (Bio-Rad, #1706516, RRID:AB_2921252, 1:1,000 dilution) was incubated with the membrane for 1 hour at room temperature and protein bands were detected by chemi-luminescent method by Pierce™ ECL Western Blotting Substrate (Thermo Fisher Scientific). The α-Actin-1 was used for normalization (mouse anti-α-Actin-1, MAB1501, RRID:AB_2223041, 1:1,000 dilution).

### 2.9 Sam68 and HPV16 E6 analysis by confocal microscopy

PCA5 cells transduced with LXSN and LXSN_E6 vector were seeded on glass bottom in 8 well chamber slide (Ibidi, Gräfelfing, Germany) for 48 h at 2.5 × 10^4^/well. Cells were fixed in 4% paraformaldehyde and permeabilized for 10 min in 1X PBS containing 0.1% TritonX-100, and incubated overnight with anti-Sam68 (Santa Cruz Biotechnology, RRID: not available, sc-514468, 1:100 dilution) or anti-HPV16 E6/18 E6 (C1P5) (Santa Cruz Biotechnology, RRID:AB_675656, sc-460, 1:50 dilution) antibodies in blocking solution (1X PBS, 0.05% TritonX-100, 5% FBS). The Anti-phalloidin Alexa Fluor 647 (Invitrogen, A22287), and 4 μM Hoechst 33342 (Thermo Fisher Scientific) were used for cytoskeleton and nuclei counterstaining, respectively. Images were acquired with a Stellaris 5 DMI8 confocal fluorescence microscope (Leica, Wetzlar, Germany) by using 63X oil immersion objective.

### 2.10 Splicing factors expression analysis in HPV-related and HPV-unrelated HNSCC

HNSCC gene expression datasets were downloaded from The Cancer Genome Atlas (TCGA) and from the Broad Institute GDAC Firehose database. Normalized RSEM (RNA-Seq by Expectation-Maximization) data from Illumina HiSeq 2000 (IlluminaHiSeq_RNASeqV2) were collected for gene expression analysis. Information about HPV infection positivity and HPV genotype were also obtained, based on TCGA RNA-Seq data analyzed by Nulton et al. (2017). Splicing factors transcripts levels were stratified according to the HPV positivity, tumor anatomical sub-site and tumor grade. Moreover, Kaplan-Meier curves were generated using KmPlot online software^2^ to investigate the prognostic value of splicing factors expression (Győrffy, 2024). Patients were assigned to low and high expression groups on the base of median gene expression value.

### 2.11 Statistical analysis

Statistical analysis was performed using GraphPad version 6 (Prism). The Pearson’s (r) and the Spearman’s (ρ) correlation coefficients were calculated for correlation analyses. The U Mann–Whitney test was used to evaluate differences in mRNA levels among sample groups. Variables with *p* ≤ 0.05 were considered statistically significant.

## 3 Results

### 3.1 Patients, HPV detection, and viral genotype characterization

The study included a cohort of 33 patients diagnosed with HNSCC and eight with HND lesions. Demographic and clinic-pathologic data for all HNSCC patients included in this study are summarized in Table 1. In particular, 72.7% (*n* = 24/33) of patients were males and 27.3% (*n* = 9/33) females, with a median age of 60 years (interquartile range, IQR: 56–67 years) and 55 years (IQR: 54–77) at diagnosis, respectively (Table 1). Most of tumors were OPSCC (57.6%, *n* = 19/33) and were poorly differentiated (G3-G4) (54.5%, *n* = 18/33) (Table 1). The search for HPV DNA was performed in all samples by broad spectrum nested PCR using MY09/11 and MGPs primer pairs followed by direct sequencing analysis of amplified products. HPV DNA was detected in 48.5% (*n* = 16/33) of HNSCC (Table 1). Stratification by anatomical sub-site showed that 47.4% (*n* = 9/19) of OPSCC, 75% (*n* = 6/8) of OCSCC and 20% (*n* = 1/5) of LSCC were positive for HPV DNA. Search for HPV DNA sequences in the GenBank database allowed to identify HPV16 as the most common viral genotype, being detected in 88.9% (*n* = 8/9), 83.3% (*n* = 5/6), and 100% (*n* = 1/1) of HPV-positive OPSCC, OCSCC, and LSCC, respectively. The HPV33 was the second most frequent genotype being detected in 11.1% (*n* = 1/9) and 16.7% (*n* = 1/6) of HPV-positive OPSCC and OCSCC, respectively.

**TABLE 1:** Clinic-pathological characteristics of patients with HNSCC enrolled in the study.

Variables	Total (*N* = 33)
**Median age at diagnosis, years (IQR)**	
Male	60 (56–67)
Female	55 (54–77)
**Sex, n (%)**	
Male	24 (72.7)
Female	9 (27.3)
**Anatomical sub-site, n (%)**	
Oropharynx	19 (57.6)
Oral cavity	8 (24.2)
Larynx	5 (15.2)
Hypopharynx	1 (3.0)
**Tumor grade, n (%)**	
G1-G2	10 (30.3)
G3-G4	18 (54.5)
Missing	5 (15.2)
**HPV DNA status, n (%)**	
Positive	16 (48.5%)
Negative	17 (51.5%)

The eight patients diagnosed with HND included six men and two women, with a median age of 50 (IQR: 33–55) and 51 (IQR: 42–60) years, respectively. HPV DNA was detected in 37.5% (*n* = 3/8) of the samples, which were all positive for HPV16.

### 3.2 HPV16 viral load, E6 full-length, and E6*I mRNAs quantification

Viral load was measured in HPV16-positive HNSCC and HND as well as in SCC152 and SCC154 cell lines, through the evaluation of HPV16 E6 DNA and TP53 copy number by ddPCR, with the formula E6/(TP53/2) to obtain the viral copy number/GE. The HPV16 load ranged from < 1 copy/GE to 27 copies/GE in HNSCC, while it was < 1 copy/GE in HND, 1 copy/GE in SCC154 and 320 copies/GE in SCC152 cell line (Supplementary Table 3).

**FIGURE 1:**
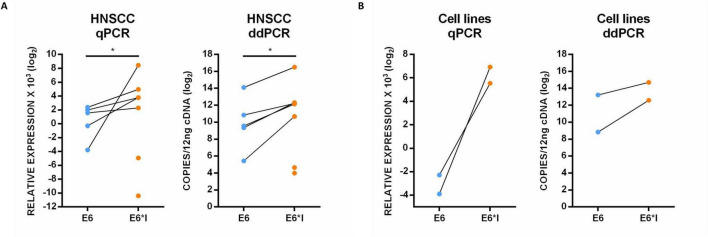
E6 and E6*I expression in HPV16-related HNSCC **(A)** as well as in SCC152 and SCC154 cell lines **(B)** analyzed by qPCR and ddPCR.

**FIGURE 2:**
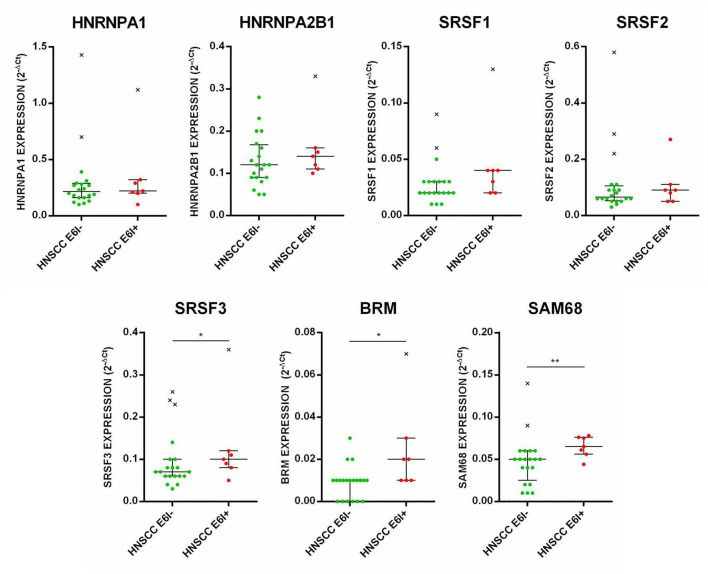
Expression profile of HNRNPA1, HNRNPA2B1, SRSF1, SRSF2, SRSF3, BRM, and SAM68 genes in E6*I-negative and positive HNSCC. Median with interquartile range is shown. **p ≤ 0.01.

The expression of E6 full-length and E6*I mRNAs were analyzed in 13 HPV16-positive HNSCC, including 7 OPSCC, 5 OCSCC, 1 LSCC, in three HPV-16 positive HND as well as in SCC152 and SCC154 cell lines either by qPCR or by ddPCR. Amplicons were visualized by polyacrilammide gel electrophoresis and subjected to direct Sanger sequencing to verify the specificity of primer pairs used for E6*I isoform amplification (Supplementary Figure 3). The E6 and E6*I transcripts were both identified in five HNSCC, with E6*I being significantly more expressed than E6 in all samples (*p* < 0.01), while only the E6*I isoform was detected in two HNSCC cases both by qPCR and ddPCR (Figure 1A). The E6 and E6*I mRNAs were both detected also in SCC152 and SCC154 cell lines, with higher levels of the E6*I isoform (Figure 2B).

In particular, the E6*I expression was observed in 71.4% (*n* = 5/7) OPSCC, 20% (*n* = 1/5) of OCSCC, and 100% (*n* = 1/1) of LSCC. Both E6 and E6*I were expressed in SCC152 and SCC154 cell lines, while no viral transcripts were detected in HND. Concordant results were obtained by analyzing E6 (ρ = 0.8, *p* = 0.001) and E6*I (ρ = 0.9, *p* < 0.001) expression with qPCR and ddPCR (Supplementary Table 4). Moreover, a statistically significant correlation was found between E6*I levels and the viral load (ρ = 0.8, *p* < 0.001) (Supplementary Table 3).

### 3.3 Expression profile of splicing factors in HNSCC and cell lines

**FIGURE 3:**
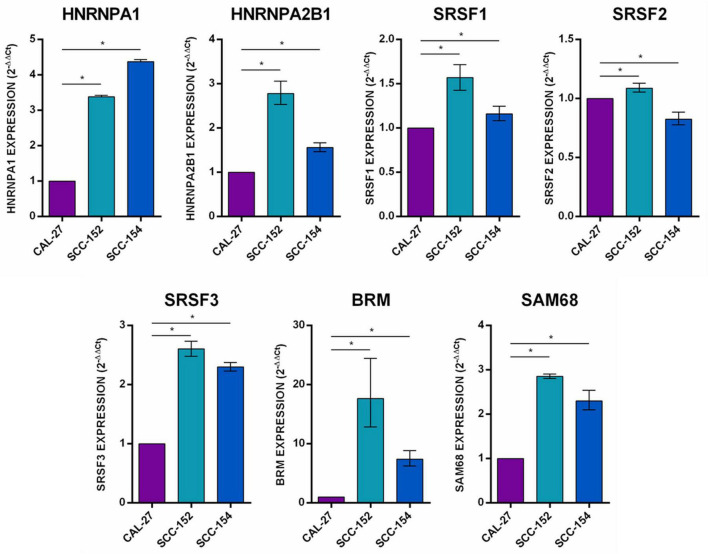
Expression analysis of splicing factors HNRNPA1, HNRNPA2B1, SRSF1, SRSF2, SRSF3, BRM, and SAM68 in CAL27, SCC152, and SCC154 cell lines. Median with interquartile range is shown. *p ≤ 0.05.

The expression levels of HNRNPA1, HNRNPA2B1, SRSF1, SRSF2, SRSF3, BRM, and SAM68 genes were analyzed in HNSCC (Figure 2) as well as in CAL27, SCC152, and SCC154 cell lines (Figure 3) by qPCR. HNSCC were stratified by HPV status, considering HPV-positive those expressing the E6*I isoform. The analysis showed a statistically significant over-expression of SRSF3, BRM and SAM68 transcripts in E6*I-positive versus E6*I-negative HNSCC (*p* < 0.5) (Figure 2).

The analysis of cell lines showed a statistically significant up-regulation of splicing factors HNRNPA1, HNRNPA2B1, SRSF1, SRSF3, BRM, and SAM68 in both SCC152 and SCC154 compared to CAL27 (Figure 3).

**FIGURE 4:**
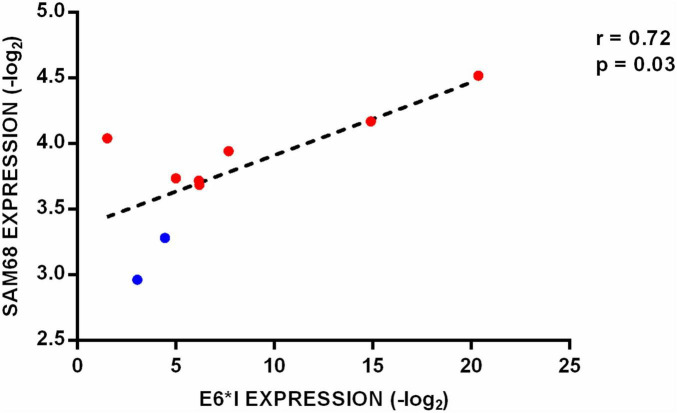
Correlation analysis between HPV16 E6*I and SAM68 expression levels in HNSCC (red dots) as well as in SCC152 and SCC154 cell lines (blue dots).

Notably, a statistically significant correlation was found between SAM68 and E6*I levels in HNSCC and cell lines (*r* = 0.72, *p* = 0.03) (Figure 4).

### 3.4 SAM68 expression in LXSN_E6 transduced PCA5 cell line

**FIGURE 5:**
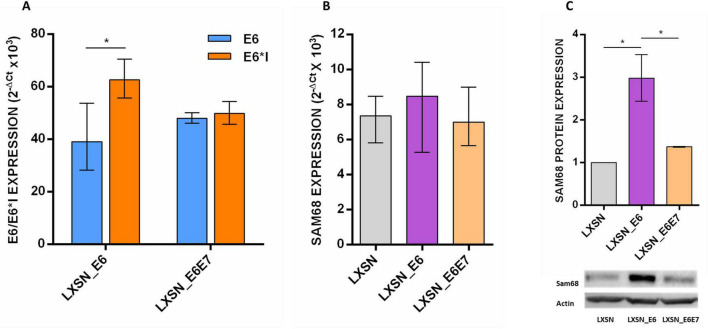
**(A)** Expression of HPV16 E6, E6*I and **(B)** SAM68 in PCA5 cell line transduced with LXSN, LXSN_E6 and LXSN_E6E7 vector; **(C)** Sam68 protein expression in transduced PCA5 cell line. Median with interquartile range is shown.

The PCA5 cell line was transduced with LXSN empty vector as well as with LXSN carrying HPV16 E6 ORF (LXSN_E6) or HPV16 E6 and E7 ORF (LXSN_E6E7). Gene expression analysis by qPCR showed higher expression of E6*I compared to E6 in cells transduced with LXSN_E6 compared to those transduced with LXSN_E6E7 (Figure 5A). Moreover, SAM68 was found significantly up-regulated in LXSN_E6-transduced PCA5 cell line at protein level and at lesser extent at mRNA level (Figures 5B,C).

**FIGURE 6:**
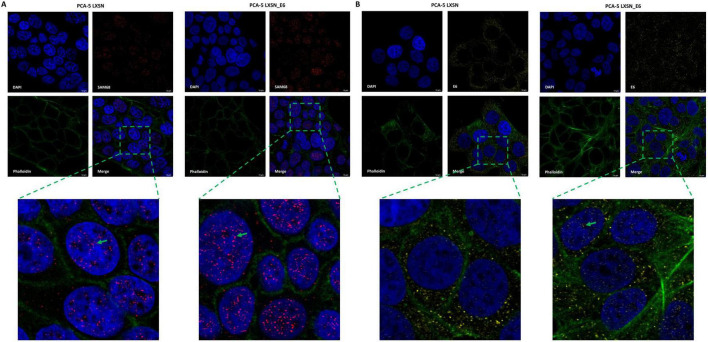
Analysis of subcellular localization of Sam68 **(A)** and HPV16 E6 oncoprotein **(B)** in PCA5 cell line transduced with LXSN or LXSN_E6 vector by confocal fluorescence microscopy. Both the proteins are indicated by green arrows.

The subcellular localization analysis of Sam68 and HPV16 E6 proteins was performed by confocal fluorescence microscopy in LXSN and LXSN_E6 transduced PCA5 cell line. The analysis showed that Sam68 localization was mainly nuclear, with increased levels in cells transduced with LXSN_E6 vector compared to those transduced with LXSN empty vector (Figure 6A). In addition, the presence of HPV16 E6 oncoprotein was confirmed in the nucleus of cells transduced with LXSN_E6 vector while no signal was observed in the nucleus of cells transduced with the LXSN empty vector (Figure 6B).

### 3.5 SAM68 silencing effects in SCC152 cell line

**FIGURE 7:**
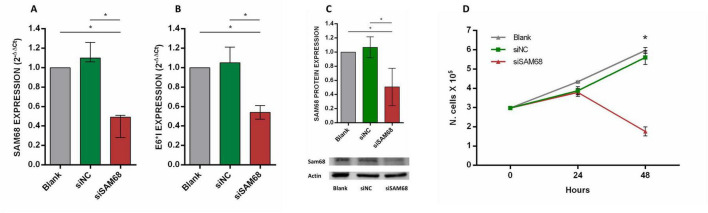
Expression analysis of SAM68 **(A)** and HPV16 E6*I **(B)** mRNAs as well as of Sam68 protein **(C)** in SCC152 cell line transfected with siRNAs targeting SAM68 (siSAM68), with negative control siRNA (siNC) and in blank cells at 48 h post-transfection; **(D)** growth curves of SCC152 cell line transfected with siSAM68, siNC and in blank cells at 48 h post-transfection. Median with interquartile range is shown. *p ≤ 0.05.

The SCC152 cell line was transfected with four siRNAs targeting SAM68 (siSAM68) or with a scrambled control siRNA (siNC). The efficacy of SAM68 silencing at 48 h post-transfection was analyzed by qPCR, showing a statistically significant reduction of SAM68 mRNA levels in cells transfected with siSAM68 compared to control and untreated cells (blank) (*p* < 0.05) (Figure 7A). A statistically significant down-regulation of HPV16 E6*I expression was also observed in cells transfected with siSAM68 (*p* < 0.05) (Figure 7B). Down-regulation of Sam68 at protein level was confirmed by western blot analysis (Figure 7C). In addition, the growth curve analysis of SCC152 cell transfected with siSAM68, with siNC and of blank cells showed a statistically significant reduction of cell number at 48 h after transfection (*p* < 0.05) (Figure 7D).

### 3.6 Analysis of splicing factors expression in HNSCC dataset

The clinical and RNA-Seq datasets of 520 HNSCC and 44 head and neck normal tissues was downloaded from TCGA and the Broad Institute GDAC Firehose databases. Normalized mRNA expression levels of HNRNPA1, HNRNPA2B1, SRSF1, SRSF2, SRSF3, BRM, and SAM68 genes were extracted and stratified by HPV status in OPSCC and OCSCC.

**FIGURE 8:**
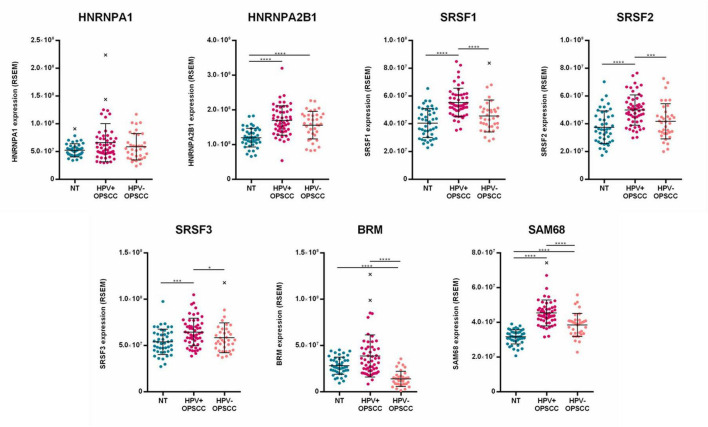
Analysis of HNRNPA1, HNRNPA2B1, SRSF1, SRSF2, SRSF3, BRM and SAM68 RNA-Seq data in OPSCC, stratified by HPV-status, as well as in normal head and neck tissues (NT) from HNSCC TCGA dataset. Median with interquartile range is shown. *p ≤ 0.05, ***p ≤ 0.001, ****p ≤ 0.0001.

The analysis of OPSCC showed a statistically significant up-regulation of HNRNPA2B1, SRSF1, SRSF2, SRSF3 and SAM68 in HPV-positive cases compared to normal tissues (*p* < 0.001). Moreover, the expression of SRSF1, SRSF2, SRSF3, BRM and SAM68 was statistically significant higher in HPV-positive versus HPV-negative cases (*p* < 0.05) (Figure 8).

**FIGURE 9:**
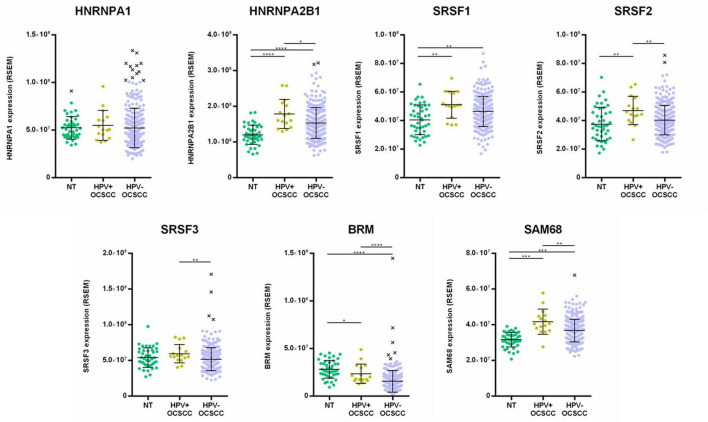
Analysis of HNRNPA1, HNRNPA2B1, SRSF1, SRSF2, SRSF3, BRM and SAM68 RNA-Seq data in OCSCC, stratified by HPV-status, as well as in normal head and neck tissues (NT) from HNSCC TCGA dataset. Median with interquartile range is shown. *p ≤ 0.05, **p ≤ 0.01, ***p ≤ 0.001, ****p ≤ 0.0001.

The analysis of OCSCC showed a statistically significant up-regulation of HNRNPA2B1, SRSF1, SRSF2, and SAM68 in HPV-positive cases compared to normal tissues (*p* < 0.01). In addition, the analysis showed that the expression of HNRNPA2B1, SRSF2, SRSF3, BRM, and SAM68 was statistically significant higher in HPV-positive versus HPV-negative OCSCC (*p* < 0.05) (Figure 9). Overall, these data confirmed the results obtained in HNSCC by qPCR.

The stratification of expression data by tumor grade revealed that HNRNPA1, HNRNPA2B1, SRSF2, SRSF3, BRM, and SAM68 levels were significantly higher in poorly differentiated/undifferentiated (G3-G4) HNSCC compared to well differentiated/moderately differentiated (G1-G2) HNSCC *(p* ≤ 0.02) (Supplementary Figure 4). Finally, the Kaplan-Meier overall survival analysis of HNSCC by KMPlot showed no prognostic values of splicing factors expression (Supplementary Figure 5).

## 4 Discussion

Many studies have demonstrated that HPV-related and HPV-unrelated HNSCC are molecularly and clinically distinct entities (Leemans et al., 2011; Sharkey Ochoa et al., 2022). For instance, patients diagnosed with HPV-related HNSCC show better prognosis, better response to treatments and higher survival rates (Fakhry et al., 2008; Ang et al., 2010; Wuerdemann et al., 2017; Ritchie et al., 2003; Li et al., 2003; Tornesello et al., 2014). Therefore, it is of primary importance to identify correctly tumors related to HPV infection in order to adopt specific and efficient therapeutic strategies.

Previous studies showed that HPV DNA is found in about 25% of OPSCC (mostly originating from tonsils), 8% of NPSCC, 7% of OCSCC, 6% of LSCC, and 4% of HPSCC (Castellsagué et al., 2016; Economopoulou et al., 2020). HPV16 is the most frequently detected genotype, being identified in 90–97% of HPV-related OPSCC (Fakhry et al., 2020; de Sanjosé et al., 2018). In the present study, HPV DNA was searched in a cohort of HNSCC as well as HND and identified in 47.4% of OPSCC, 75% of OCSCC, 20% of LSCC, and 37.5% of HND. In agreement with previous studies, HPV16 was the most frequent viral genotype both in HNSCC and HND.

The viral load was analyzed in all HPV16-positive samples and found to range between < 1 copy/GE to 27 copies/GE in HNSCC, while less than 1 copy/GE was found in HND. Such variability has been also reported in other studies, although the different methods used to quantify the viral copy number do not allow an accurate comparison of results (Veitía et al., 2020; Zhang et al., 2010). Several studies investigated the role of HPV viral load as prognostic biomarker in HNSCC, however its efficacy is still debated (Hashida et al., 2021; Mena et al., 2018; Stevenson et al., 2020).

The detection of HPV DNA sequences alone does not distinguish transient infections, which are unrelated to tumor development, from oncogenic infections, characterized by the presence transcriptionally active HPV (Boscolo-Rizzo et al., 2016; Holzinger et al., 2012). Indeed, the expression of E6*I mRNA has been proposed as a reliable biomarker of transforming HPV infection, with greater sensitivity compared to E6 and viral DNA (Shi et al., 2009; Westra, 2014; Masterson et al., 2016; Mena et al., 2022). For example, de Sanjosé et al., showed that HPV DNA was detected in 24.9, 7.4, and 5.7% of analyzed OPSCC, OCSCC and LSCC, respectively, while E6*I expression was identified in 22.4, 4.4, and 3.5% of cases, respectively (de Sanjosé et al., 2018).

In the present study, the E6*I was found expressed in 71.4% (*n* = 5/7) of OPSCC, 20% (*n* = 1/5) of OCSCC and 100% (*n* = 1/1) of LSCC, while no viral transcripts were detected in HPSCC and HND. Such results indicate that in several cases the presence of HPV DNA is related to an active viral infection. Overall, the HPV16 E6*I levels showed a statistically significant correlation with viral load (Hashida et al., 2021; Deng et al., 2013; Jung et al., 2010).

Splicing factors activity was found deregulated in different tumor types, causing the production of oncogenic mRNA isoforms which affect all cancer hallmarks (Dvinge et al., 2016). The alternative splicing of the E6/E7 pre-mRNA is mediated by the coordinated activity of splicing factors that enhance or inhibit splicing reactions during carcinogenesis, however the mechanisms underlying the increased E6*I production in tumors are not clear. Hence, characterizing such splicing factors may be useful to identify new tumor progression biomarkers and potential therapeutic targets (Stanley and Abdel-Wahab, 2022; Bonnal et al., 2020). To date, very few studies investigated the deregulation of splicing factors and their role in HPV16 E6/E7 pre-mRNA maturation in HNSCC. For example, Yu et al. demonstrated that hnRNPA1 is up-regulated in OCSCC, stimulating cell proliferation by promoting exon 5 inclusion in cyclin-dependent kinase 2 (CDK2) mRNA (Yu et al., 2015). HnRNPA2B1 was found overexpressed in OCSCC, altering the N6-methyladenosine methylation pattern in different transcripts, which are associated with cancer progression, metastasis and worse prognosis (Pan et al., 2023; Zhu et al., 2021). Specifically, abnormal hnRNPA2B1 expression was found to induce the epithelial-mesenchymal transition (EMT) process by increasing the activity of the LINE-1/TGF-β1/Smad2/Slug signaling pathway (Zhu et al., 2021). In addition, Zhang et al. showed that SRSF1 is overexpressed in OCSCC and binds to lncRNA LINC01296, promoting cell proliferation, invasion and EMT (Zhang et al., 2021). It was also demonstrated that SRSF1 overexpression is associated with poor outcome in OCSCC patients and that its siRNA-mediated knockdown in CAL27 and SCC4 cell lines inhibited lysosomal biogenesis and enzyme activity, cell growth and proliferation and xenograft growth in mice (Qu et al., 2023). Similarly, SRSF3 was also found overexpressed in OCSCC, particularly in high-grade tumors, inducing EMT by Slug and N-cadherin up-regulation, and inhibiting autophagy by p65 and FOXO1 down-regulation (Peiqi et al., 2016; Guo et al., 2015; Zhou et al., 2019).

In this study, the expression profile of HNRNPA1, HNRNPA2B1, SRSF1, SRSF2, SRSF3, BRM, and SAM68 was analyzed in HNSCC as well as in CAL27, SCC152, and SCC154 by qPCR. The analysis showed that splicing factors SRSF3, BRM and SAM68 were up-regulated in E6*I-positive compared to E6*I-negative HNSCC (*p* < 0.05). Moreover, HNRNPA1, HNRNPA2B1, SRSF1, SRSF3, BRM, and SAM68 were significantly up-regulated both in SCC152 and SCC154 cell lines compared to CAL27 cell lines (*p* < 0.05). Overall, such results were confirmed by the analysis of RNA-Seq data from the HNSCC cohort present in the TCGA database and suggest that HPV16 has a role in splicing factors deregulation in HNSCC. In addition, SAM68 levels were found to correlate with E6*I expression (*r* = 0.7, *p* = 0.03). This data suggest that HPV may drive splicing factors up-regulation in HNSCC and that SAM68 may play an important role in HPV16 E6*I mRNA production.

To confirm the role of HPV16 in SAM68 deregulation, HPV16 E6 was introduced in a HPV-negative SCC-derived cell line and oncoviral transcripts as well as Sam68 quantified. The analysis showed increased production of E6*I mRNA and higher expression of Sam68 mostly at protein level and at a lesser extent at transcriptional level in cells transduced with LXSN_E6. These data suggest that HPV16 E6 plays a role in Sam68 up-regulation mainly at post-transcriptional level in HNSCC. Indeed, it has been shown that the E6 oncoprotein is able to modulate gene expression with post-transcriptional mechanisms (Billingsley et al., 2022). In particular, the HPV16 E6 has been demonstrated to interact with the longer splice variant of nuclear transcription factor, X-Box binding 1, namely NFX1-123, together with E6AP, favoring the recruitment of cytoplasmic poly (A) binding proteins (PABPCs) on TERT mRNA, causing increased stabilization of TERT transcript and enhanced activity of telomerase in human keratinocytes (Katzenellenbogen et al., 2009; Tornesello et al., 2022). Based on these studies, we may speculate that the HPV16 E6 is able to stabilize SAM68 transcript, with consequent increased protein production.

Sam68 is considered the prototypic member of the signal transduction and activation of RNA (STAR) family of RNA-binding proteins (RBPs), involved in cell signaling in response to extracellular stimuli, RNA transcription, splicing and nuclear export (Sánchez-Jiménez and Sánchez-Margalet, 2013; Najib et al., 2005; Taylor and Shalloway, 1994). It was demonstrated that Sam68 localizes in nuclear speckles, namely Sam68 nuclear bodies (SNBs), together with RNA molecules, splicing factors YT521-B and hnRNP L and two Sam68-like mammalian proteins, SLM1 and SLM2 (Chen et al., 1999; Rajan et al., 2009; Hartmann et al., 1999). In addition, proteomic analysis demonstrated that Sam68 is able to form small or large ribonucleoprotein complexes, composed of several RNA binding proteins, with an EGF-dependent mechanism. These two complexes exist in equilibrium in transformed cell lines, with the smaller one being responsible for Sam68 splicing regulation activity (Huot et al., 2009). In this study, the immunofluorescence analysis of HPV-negative PCA5 cell line transduced with LXSN_E6 vector confirmed the presence of HPV16 E6 oncoprotein in the nucleus of the cells and showed increased nuclear expression of Sam68 compared to control cells. These results support the hypothesis that the E6 oncoprotein stabilizes Sam68 in the nucleus. However, further studies are needed to elucidate the type of mechanism involved in Sam68 and E6 interaction.

To further validate the interplay between SAM68 and HPV16 E6*I we performed silencing experiment by using siRNAs specifically targeting SAM68 in SCC152 cell line. The results showed that SAM68 down-regulation caused reduced expression of E6*I, confirming the role of SAM68 in E6*I production in HNSCC.

Sam68 was found up-regulated and associated with poor prognosis in different cancer types, including hepatocellular, gastric, renal, prostate, ovarian, breast, and non-small cell lung carcinoma, however a very few studies investigated the role of Sam68 in HNSCC (Busà et al., 2007; Zhang et al., 2009; Zhang et al., 2014; Zhang et al., 2015; Paronetto et al., 2010; Dong et al., 2016). For example, it has been shown that Sam68 over-expression promotes LNCaP prostate cancer cell lines proliferation and resistence to DNA-damaging agents, such as cisplatin (Busà et al., 2007). Moreover, the siRNA-mediated silencing of SAM68 reduced proliferation and colony formation of OVCAR-3 ovarian cancer cell line (Dong et al., 2016). Similarly, the up-regulation of Sam68 in MCF-7 and MDA-MB-231 breast cancer cell lines correlates with enhanced cell proliferation through activation of Akt/GSK-3β signaling (Song et al., 2010). Recently, Komiyama et al. demonstrated that Sam68 is overexpressed and associated with poor prognosis in OCSCC, and that its knockdown in HO-1N-1 cells reduced vimentin expression and cell motility (Komiyama et al., 2022). Sam68 up-regulation was also shown to induce the expression of the anti-apoptotic proteins caspase-9, caspase-3 and PARP, inhibiting cisplatin-induced apoptosis in oral tongue SCC-derived SCC-9 and SCC-25 cell lines (Chen et al., 2016). In this study, silencing of SAM68 in SCC152 cell line caused statistically significant inhibition of cell growth, confirming the oncogenic role of SAM68 in HNSCC.

Finally, the analysis of RNA-Seq data from TCGA datasets confirmed the up-regulation of splicing factors SRSF3, BRM and SAM68 in HPV-positive HNSCC. In addition, the expression of splicing factors was higher in G3-G4 compared to G1-G2 tumors, suggesting their association with cancer progression in HNSCC. Therapies targeting splicing factors may be relevant to prevent tumor evolution. For example, treatment of CAL-9 OCSCC cell line with SRSF3-specific antisense oligonucleotide was shown to reduce cell growth and sensitized cells to paclitaxel (Sun et al., 2019).

## 5 Conclusion

In conclusion, the present study showed that splicing factors SRSF3, BRM and SAM68 genes are up-regulated in E6*I-positive HNSCC, suggesting a role for HPV in splicing deregulation. Specifically, HPV16 E6 and E6* proteins may contribute to the over-expression of SAM68, which in turn may have a major role in the production of E6*I isoform. In addition, SAM68 over-expression was found to contribute to the oncogenic phenotype of HNSCC-derived SCC152 cell line, promoting cell proliferation. Further analyses are needed to confirm such data and to investigate the role of SAM68 as a potential novel therapeutic target in HPV-related HNSCC.

## Data Availability

The datasets presented in this study can be found in online repositories. The names of the repository/repositories and accession number(s) can be found here: https://zenodo.org/records/18231290.

## Footnotes

1
http://www.ncbi.nlm.nih.gov/blast

2
https://kmplot.com/
