# Supplementary material for: Human papillomavirus 16 E6 oncoprotein promotes up-regulation of RNA-binding protein Sam68 in head and neck cancer
Source: Front Microbiol. 2026 Feb 5;17:1657818. doi: 10.3389/fmicb.2026.1657818 (PMC12916563; doi:10.3389/fmicb.2026.1657818)
Supplement: Supplementary file 1 [file Presentation_1.pdf]

Supplementary Material

1 Supplementary Figures and Tables

1.1 Supplementary Figures

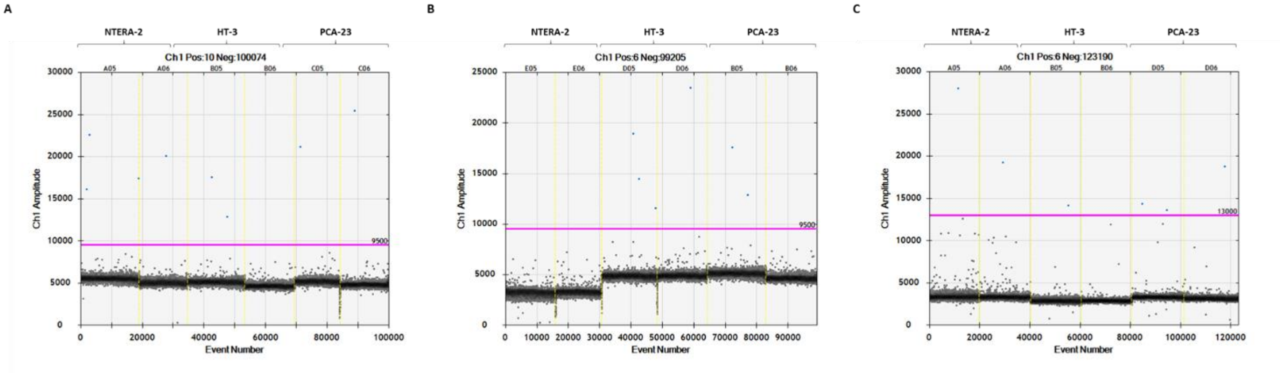

Supplementary figure 1. (A) Limit of blank (LOB) for HPV16 E6 gene amplification was assessed by amplifying HPV-negative NTERA-2, HT-3, and PCA-23 cell lines DNA samples. (B, C) LOB for HPV16 E6 and E6*I transcripts, respectively, was assessed by amplifying the viral mRNAs in HPV-negative NTERA-2, HT-3, and PCA-23 cell lines cDNA samples. False-positive droplets mean ± standard deviation was calculated. The observed LOB for E6 was 1.3 ± 1 both on DNA and cDNA samples, while the LOB for E6*I was 1 ± 0.6.

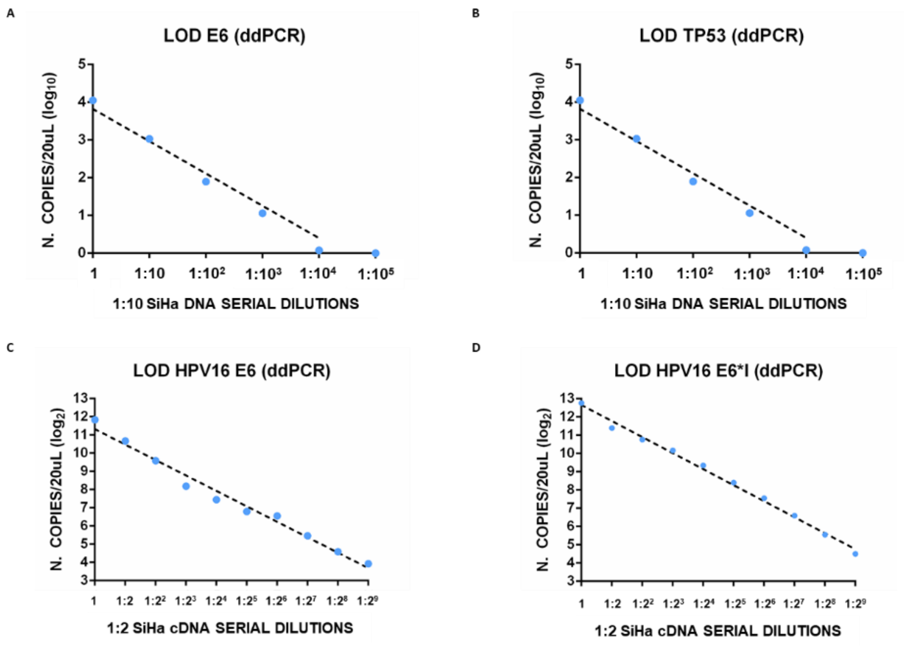

Supplementary figure 2. (A) Limit of detection (LOD) for HPV16 E6 gene and (B) TP53 gene was determined by amplification of 1:10 serial dilutions of HPV16-positive SiHa cell line DNA (1 – 1:100’000, corresponding to 100ng – 0.001ng DNA). Observed LOD was = 1 absolute copy. (C) LOD for HPV16 E6 and (D) E6*I transcripts was determined by amplification of 1:2 serial dilutions of SiHa cell line cDNA (1 – 1:512, corresponding to 25ng – 0.05ng cDNA) into HPV-negative NTERA-2 cell line cDNA. Observed LOD for both E6 and E6*I was <20 absolute copies.

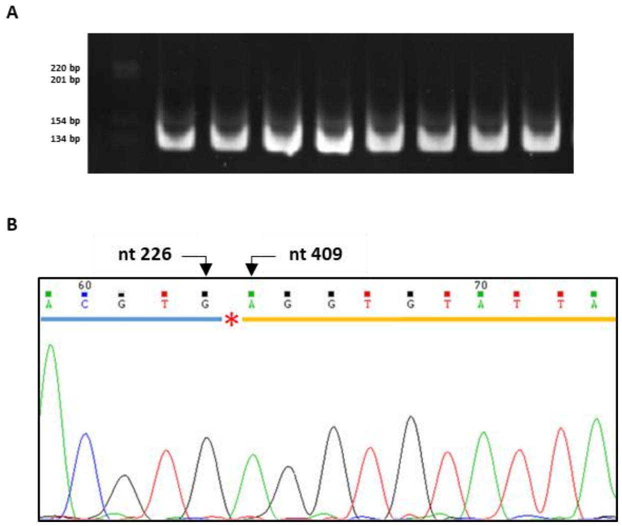

Supplementary figure 3. (A) Electrophoresis and (B) sequencing analysis of HPV16 E6*I amplicons. Splice donor (nt 226) and splice acceptor (nt 409) sites are indicated in the electropherogram; exons are underlined in blue and yellow; the exons joining site is indicated as a red star.

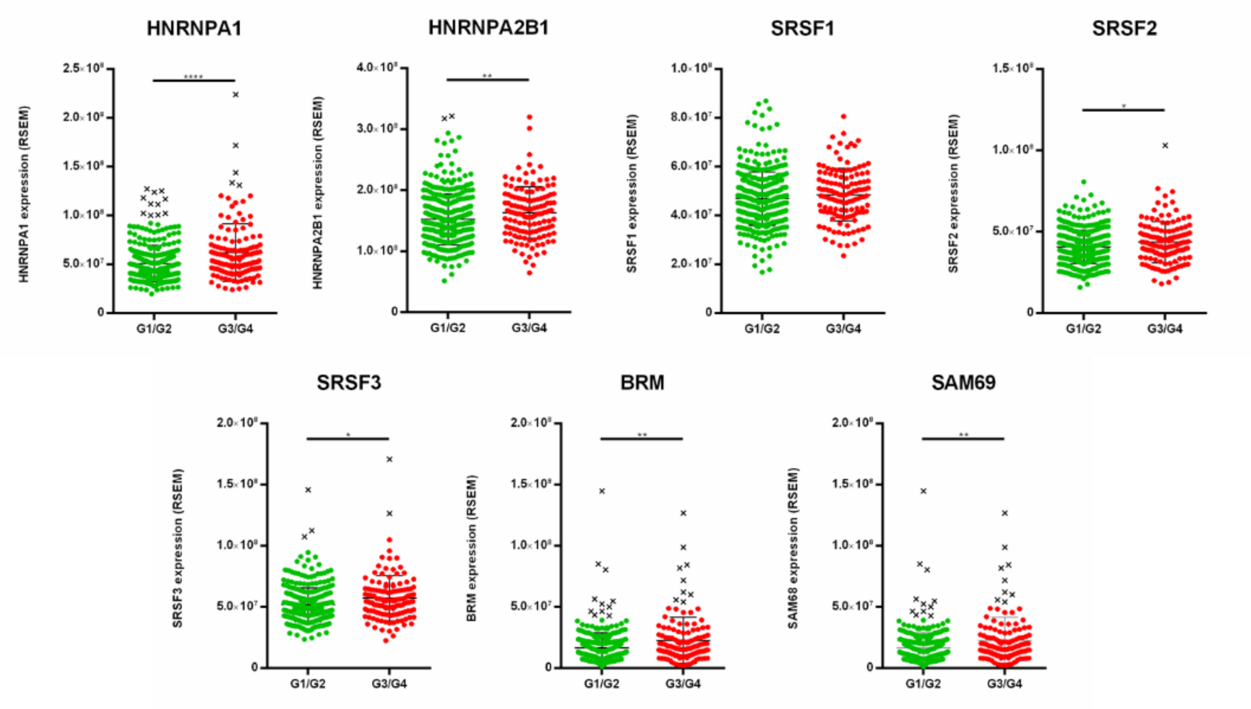

Supplementary figure 4. RNA-Seq data analysis of HNRNPA1, HNRNPA2B1, SRSF1, SRSF2, SRSF3, BRM and SAM68 transcripts from HNSCC TCGA dataset, stratified by tumor grade. Outlier values (x) were cleared for the analysis.

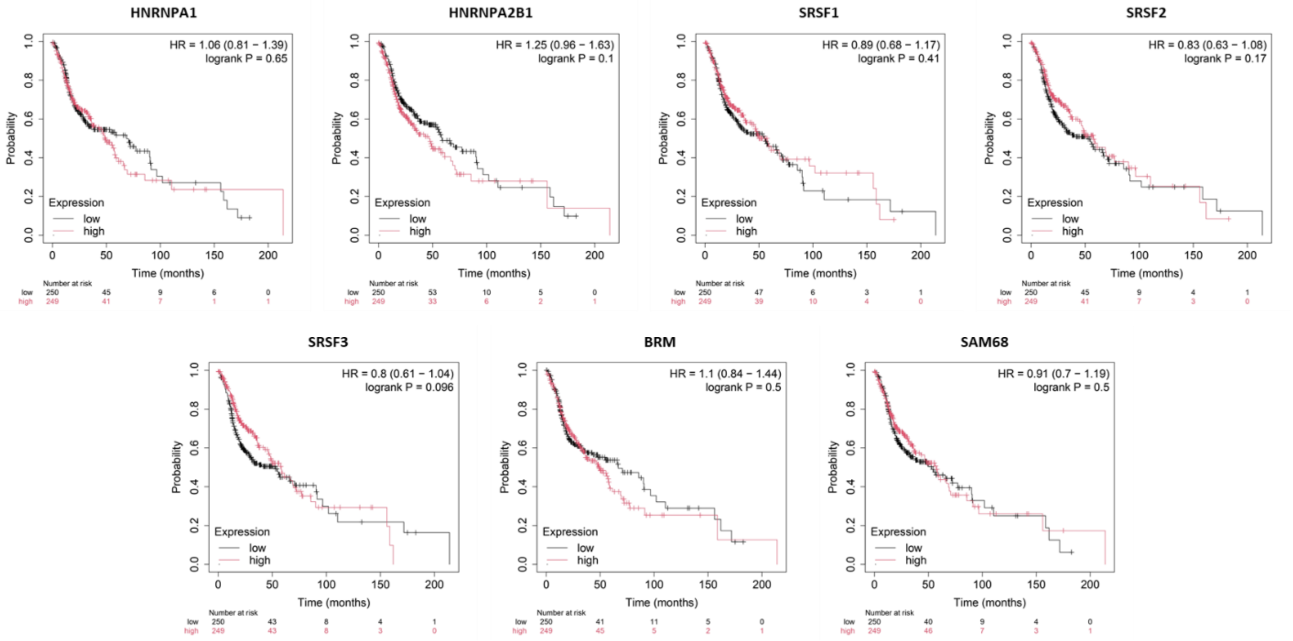

Supplementary figure 5. Kaplan-Meier overall survival analysis of HNSCC cases from TCGA dataset. Patients were stratified by HNRNPA1, HNRNPA2B1, SRSF1, SRSF2, SRSF3, BRM and SAM69 median expression values. The analysis was performed by KMPlot online software.

1.2 Supplementary Tables

Supplementary Table 1. Primer sequences used to amplify HPV16 E6 and E6*I as well as HNRPA1, HNRNPA2B1, SRSF1, SRSF2, SRSF3, BRM, SAM68, TP53, GAPDH and ACTB.

Locus	Primers name	Sequences (5'-3')	Amplicon Size (bp)	Ta (°C)	References
HPV16 E6	HPV16RNAF1F2<br>HPV16RNAR1	AAGTTACCACAGTTATGC<br>TGTTCTAATGTTGTTCCAT	239	60.0	This study
HPV16 E6*I	HPV16E6sI-IIFor<br>HPV16E6sIRev	AGGAGCGACCCAGAAAGTTA<br>GCTTTTGACAGTTAATACACCTCAC	107	60.0	Pastuszak-Lewandoska, 2014
HNRNPA1	HNRNPA1-For<br>HNRNPA1-Rev	TGGATTTGGTAATGATGGAA<br>TCTCTGGCTCTCCTCTCCTG	267	60.0	Park et al, 2016
HNRNPA2B1	HNRNPA2B1-For<br>HNRNPA2B1-Rev	AGCTTTGAAACCACAGAAGAA<br>TTGATCTTTTGCTTGCAGGA	100	60.0	Brandi et al, 2016
SRSF1	SRSF1-For<br>SRSF1-Rev	TGCCTACATCCGGGTTAAAG<br>CTGCTGTTGCTTCTGCTACG	105	60.0	Park et al, 2016
SRSF2	SRSF2-For<br>SRSF2-Rev	CGAAGAAGAGTCCTTGGA<br>AAGTCGTTCACCTCACTAA	167	54.8	This study
SRSF3	SRSF3-For<br>SRSF3-Rev	GCATCGTGATTCCTGTCCAT<br>AATTCCGTCTTGTTGCCATTG	75	54.8	This study
BRM	BRM-For<br>BRM-Rev	AGATTCTGTATCGCCATA<br>GCATAATAGTGTTCATAAGTG	108	55.5	This study
SAM68	SAM68-For<br>SAM68-Rev	AGGCTTATGCTCTTATGG<br>AATTGCTCCTGACAGATAT	88	54.8	This study
TP53	P53ex7a<br>P53ex7b	TCTCCTAGGTTGGCTCTGAC<br>CAAGTGGCTCCTGACCTGGA	134	60.0	This study
GAPDH	GAPDH-Fw<br>GAPDH-Rev	TGACAACAGCCTCAAGATCATC<br>GTCTTCTGGGTGGCAGTGAT	141	60.0	This study
ACTB	ACTB-Fw<br>ACTB-Rev	CTGGAACGGTGAAGGTGACA<br>ACTTCCTGTAACAACGCATCTCA	135	60.0	This study

Supplementary Table 2. Checklist of Minimum Information for Publication of Digital PCR Experiments 2020 (dMIQE) for HPV16 load as well as HPV16 E6 and E6*I transcripts quantification.

ITEM TO CHECK	PROVIDED	COMMENT
	Y/N	
1. SPECIMEN		
Detailed description of specimen type and numbers	Y	HPV16 load: fresh frozen biopsies of head and neck squamous cell carcinoma (n=14) and dysplasia (n=3).<br>HPV16 E6 and E6*I: fresh frozen biopsies of head and neck squamous cell carcinoma (n=13) and dysplasia (n=3).
Sampling procedure (including time to storage)	Y	At the time of resection each fresh frozen biopsy was immediately submerged in liquid nitrogen.
Sample aliquotation, storage conditions and duration	Y	Liquid nitrogen until analysis.
2. NUCLEIC ACID EXTRACTION		
Description of extraction method including amount of sample processed	Y	DNA: 10mg of tissue were digested with proteinase K (150 µg/ml) in 500µL of lysis buffer (10mM Tris-HCL, pH 7.6, 5mM EDTA, 150mM NaCl, 1% SDS) at 37°C, over-night.

		DNA was purified with phenol-chloroform-isoamyl alcohol (25:24:1) and precipitated with 0.3M sodium acetate (pH 4.6) in ethanol. RNA: 25-30mg of tissue were dissociated with gentleMACS Octo Dissociator (Miltenyi Biotec, Bergisch Gladbach, Germany). Then, RNA was isolated by using RNeasy MiniKit (Qiagen, Hilden, Germany) according to manufacturer procedure.
Volume of solvent used to lute/resuspend extract	Y	DNA: 50-100 µL TE. RNA: 30 µL nuclease-free water.
Number of extraction replicates	Y	1-2
Extraction blanks included?	Y	Extraction of blanks was included every 5 samples.
3. NUCLEIC ACID ASSESSMENT AND STORAGE		
Method to evaluate quality of nucleic acids	Y	See below.
Method to evaluate quantity of nucleic acids (including molecular weight and calculations when using mass)	Y	Quantity of DNA and RNA samples was determined by Nanodrop 2000c spectrophotometer (Thermo Fisher Scientific, Waltham, Massachusetts). Quality was assessed by the ratio of absorbance at 260 nm and 280 nm required to be above 1.8.
Storage conditions: temperature, concentration, duration, buffer, aliquots	Y	3-5 aliquots of each DNA and RNA sample (50-500ng/µL TE) were stored at -80°C for two years.
Clear description of dilution steps used to prepare working DNA solution	Y	Working DNA and RNA solutions (50-500ng/µL) is obtained by calculating the dilution factor ([DNA/RNA]ng/50ng) and by adding the stock DNA or RNA to the required TE and nuclease-free water volume, respectively.
4. NUCLEIC ACID MODIFICATION		
Template modification (digestion, sonication, pre-amplification, bisulphite etc.)	N	
Details of repurification following modification if performed	N	
5. REVERSE TRANSCRIPTION		
cDNA priming method and concentration	Y	cDNA priming was performed by using a combination of oligo(dT) and random primers. cDNA final concentration was 12.5ng/µL.
One or two step protocol (include reaction details for two step)	Y	Two step protocol:<br>1. 25°C for 5min<br>2. 46°C for 20min<br>3. 95°C for 1min<br>4. Holding at 4°C.
Amount of RNA added per reaction	Y	250ng
Detailed reaction components and conditions	Y	See Materials and Methods.
Estimated copies measured with and without addition of RT*	N	
Manufacturer of reagents used with catalogue and lot numbers	Y	iScript cDNA Synthesis Kit, 100 x 20 µl rxns (cat. 1708891, lot. 64570510), Bio-Rad
Storage of cDNA: temperature, concentration, duration, buffer and aliquots	Y	Two aliquots per sample of cDNA (12.5 ng/µl in a final volume of 20µL nuclease-free water) were stored at -20°C until analysis.
6. ddPCR PRIMERS DESIGN AND TARGET INFORMATION		

Sequence accession number or official gene symbol	Y	HPV16 E6; HPV16 E6*I; TP53.
Method (software) used for design and *in silico* verification	Y	Primers for HPV16 E6 and TP53 were designed and verified with the software Beacon Designer (Bio-Rad, Hercules, California); primers for HPV16 E6*I were from Pastuszak-Lewandoska, 2014 (see Supplementary Table 2)
Location of amplicon	Y	5'-nucleotide position for HPV16 E6: 134 (Forward), 372 (Reverse).<br>5'-nucleotide position for HPV16 E6*I: 120 (Forward), 428 (Reverse).<br>5'-nucleotide position for TP53: 783 (Forward), 651 (Reverse).<br>Nucleotide positions of primers are given on the GenBank sequence number K02718.1 (HPV16 E6 and E6*I) and JQ751553.1 (TP53).
Amplicon length	Y	HPV16 E6: 239bp; HPV16 E6*I: 107bp; TP53: 134bp
Primer and probe sequences (or amplicon context sequence)**	N	See Aupplementary table 2.
Location and identity of any modifications	N	
Manufacturer of oligonucleotides	Y	Eurofins Genomics GmbH (Ebersberg, Germany).
7. ddPCR PROTOCOL		
Manifacturer of ddPCR instrument and instrument model	Y	QX200 Digital droplet PCR System (Bio-Rad)
Buffer/Kit manufacturer with catalogue and lot number	Y	QX200 ddPCR EvaGreen Supermix (cat. 1864033, lot. 64494187)
Primer and probe concentration	Y	See Materials and Methods
Pre-reaction volume and composition (incl. amount of template and if restriction enzyme added)	Y	See Materials and Methods
Template treatment (initial heating or chemical denaturation)	N	
Polymerase identity and concentration, Mg++ and dNTP concentrations***	N	
Complete thermocycling parameters	Y	HPV16 E6 and E6*I:<br>1. Enzyme activation: 5 min 95°C<br>2. 40 cycles of 30 sec denaturation at 95°C and 1 min annealing/extension at 52°C<br>3. Signal stabilization at 4°C for 5 min and 90°C for 5 min<br>4. Holding at 4°C<br>TP53:<br>1. Enzyme activation: 5 min 95°C<br>2. 40 cycles of 30 sec denaturation at 95°C and 1 min annealing/extension at 60°C<br>3. Signal stabilization at 4°C for 5 min and 90°C for 5 min<br>4. Holding at 4°C
8. ASSAY VALIDATION		
Details of optimization performed	Y	See Materials and Methods
Analytical specificity (vs. related sequences) and limit of blank (LOB)	Y	Supplementary Figure 1
Analytical sensitivity/LOD and how this was evaluated	Y	Supplementary Figure 2

Testing for inhibitors (from biological matrix/extraction)	N	
9. DATA ANALYSIS		
Description of ddPCR experimental design	Y	See Materials and Methods
Comprehensive details negative and positive of controls (whether applied for QC or for estimation of error)	Y	Supplementary figure 1
Partition classification method (thresholding)	Y	Manual
Examples of positive and negative experimental results (including fluorescence plots in supplemental material)	Y	Supplementary Figure 1
Description of technical replication	N	
Repeatability (intra-experiment variation)	Y	Supplementary Figure 1, Supplementary Figure 2
Reproducibility (inter-experiment/user/lab etc. variation)	Y	Supplementary Figure 1, Supplementary Figure 2
Number of partitions measured (average and standard deviation)	Y	17.100 (±1526)
Partition volume	N	
Copies per partition (λ or equivalent) (average and standard deviation)	N	
ddPCR analysis program (source, version)	Y	See Materials and Methods
Description of normalisation method	Y	See Materials and Methods
Statistical methods used for analysis	Y	See Materials and Methods
Data transparency	raw data available on request	m.tornesello@istitutotumori.na.it

* Assessing the absence of DNA using a no RT assay (or where RT has been inactivated) is essential when first extracting RNA. Once the sample has been validated as DNA-free, inclusion of a no-RT control is desirable, but no longer essential.
** Disclosure of the primer and probe sequence is highly desirable and strongly encouraged. However, since not all commercial pre-designed assay vendors provide this information when it is not available assay context sequences must be submitted (Bustin et al. Primer sequence disclosure: A clarification of the MIQe guidelines. Clin Chem 2011;57:919-21.)
*** Details of reaction components is highly desirable, however not always possible for commercial disclosure reasons. Inclusion of catalogue number is essential where component reagent details are not available.

Supplementary Table 3. HPV16 viral copy number/genome equivalent (GE) in HNSCC, HND and cell lines. The p-value of correlation analysis between viral load and E6*I expression is reported.

Samples	Anatomical sub-site	HPV16 copies/GE (ddPCR)	E6*I copies/12ng cDNA (ddPCR)	p-value
OPSCC-1	Oropharynx	<1	0.0	<0.001
OPSCC-2	Oropharynx	8.0	4740.0	
OPSCC-4	Oropharynx	2.6	NA	
OPSCC-14	Oropharynx	<1	0.0	
OPSCC-15	Oropharynx	2.7	5090.0	
OPSCC-17	Oropharynx	<1	14.2	
OPSCC-18	Oropharynx	26.7	1637.0	
OPSCC-19	Oropharynx	10.7	91900.0	
OCSCC-1	Oral cavity	<1	0.0	
OCSCC-2	Oral cavity	<1	0.0	
OCSCC-3	Oral cavity	<1	0.0	
OCSCC-4	Oral cavity	4.8	4550.0	
OCSCC-7	Oral cavity	<1	5.3	
LSCC-5	Larynx	<1	9.2	
OPD-2	Oropharynx	<1	0.0	
OPD-4	Oropharynx	<1	0.0	
OCD-1	Oral cavity	<1	0.0	
SCC-152	Hypopharynx	320.5	26580.0	
SCC-154	Oral cavity	1	6120.0	

HNSCC = head and neck SCC, HND = head and neck dysplasia, OPSCC = oropharyngeal SCC, OCSCC = oral cavity SCC, LSCC = laryngeal SCC, OPD = oropharyngeal dysplasia, OCD = oral cavity dysplasia.

Supplementary table 4. HPV16 E6 and E6*I isoform expression levels detected by qPCR and ddPCR in OPSCC, CSCC, LSCC and cell lines. A total of 12ng cDNA were analysed by both qPCR and ddPCR. The p-value of correlation analysis between qPCR and ddPCR results is reported.

Sample ID	qPCR[a]	ddPCR[b]		qPCR[a]	ddPCR[b]	
	E6	E6	p-value	E6*I	E6*I	p-value
OPSCC-1	0.00	0.00	0.001	0.00	0.00	<0.001
OPSCC-2	2.99	1830.00		4.91	4740.0	
OPSCC-14	0.00	0.00		0.00	0.00	
OPSCC-15	5.20	652.00		31.45	5090.0	
OPSCC-17	0.00	0.03		0.00	25.00	
OPSCC-18	0.82	43.00		13.93	1637.00	
OPSCC-19	0.07	17490.00		351.06	91900.00	
OCSCC-1	0.00	0.00		0.00	0.00	
OCSCC-2	0.00	0.00		0.00	0.00	
OCSCC-3	0.00	0.00		0.00	0.00	
OCSCC-5	4.01	754.00		13.67	4550.00	
OCSCC-8	0.00	0.00		0.00	0.00	
LSCC-5	0.00	0.00		0.00	16.00	
SCC-152	0.07	9440.00		121.59	26580.00	
SCC-154	0.21	455.00		46.34	6120.00	

a = expression reported as $2^{-\Delta Ct}$ X 10^3, b = expression reported as copies/12ng cDNA, OPSCC = oropharyngeal SCC, OCSCC = oral cavity SCC, LSCC = laryngeal SCC.
